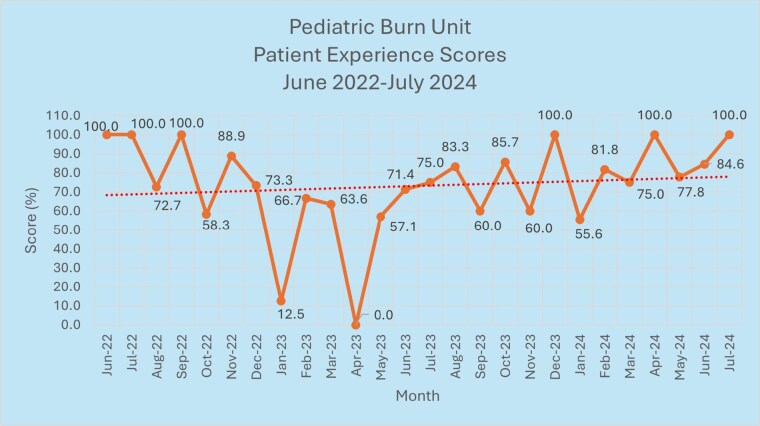# 669 Intentional Rounding and Focused Questioning to Increase Patient Satisfaction Scores on a Pediatric Burn Unit

**DOI:** 10.1093/jbcr/iraf019.298

**Published:** 2025-04-01

**Authors:** Lisa Shostrand, Sharon Albright, Brett Hartman

**Affiliations:** Riley Hospital for Children; Riley Hospital for Children; Eskenazi Health

## Abstract

**Introduction:**

Favorable patient satisfaction scores can be indicative of quality care and positive patient experiences, fostering trust between healthcare providers and patients. A focus on patient satisfaction encourages healthcare professionals to continually assess and improve their services, fostering a culture of excellence and continuous improvement. Based on feedback trends, members of the Pediatric Burn Unit seized an opportunity to redesign patient rounding standard work to enhance patient and family experiences.

**Methods:**

Patient feedback surveys from June 2022 to June 2023 displayed a 69.6% experience score overview. Pediatric Burn Unit team members created a workgroup to assess trends in patient satisfaction scores and collaborate on strategies to enhance overall improvement. Beginning in June 2023, the workgroup established action items to address deficiencies. Such tactics included comprehensive unit staff and leadership rounding, asking families “what matters most” each shift, additional room signage, team member recognition programs, and incorporating team member performance development goals surrounding patient experience.

**Results:**

After implementation, overall averages from July 2023 to July 2024 yielded 80.4%, a net overall gain of 10.8%. Trends continued an upward trajectory. All Pediatric Burn Unit team members, including leadership, therapy, and bedside staff, routinely rounded and prioritized patient and family questions and concerns.

**Conclusions:**

A positive relationship between the addition of multilayer patient rounding, visual management, and staff member ownership of the patient satisfaction paradigm was appreciated. Consistent and repeated interaction with Pediatric Burn Unit staff and other various medical teams with families resulted in enhanced patient satisfaction scores with upward progression.

**Applicability of Research to Practice:**

Consistent patient interactions and focused questioning are fundamental components in the realm of patient satisfaction improvement. By systematically addressing patients’ key concerns, Pediatric Burn Unit staff can develop an understanding of patient experiences, ultimately leading to more targeted and effective interventions and outcomes.

**Funding for the Study:**

N/A